# Evaluating the presidentially mandated accelerated expansion of the VA Veteran-Directed Care program

**DOI:** 10.1093/geroni/igaf122.4110

**Published:** 2025-12-31

**Authors:** Krysttel Stryczek, Wendy Hathaway, Stuti Dang, Leah Haverhals

**Affiliations:** VA Eastern Colorado Healthcare System, Aurora, Colorado, United States; Transformative Health Systems Research to Improve Veteran Equity and Independence (THRIVE) Center of Innovation, VA Providence Healthcare System, Providence, Rhode Island, United States; Miami VA Healthcare System, Miami VA Healthcare System, Florida, United States; VA Eastern Colorado Healthcare System, Aurora, Colorado, United States

## Abstract

The 2023 United Sates Presidential Executive Order 14095 mandated the Veterans Health Administration’s (VA) expansion of the Veteran-Directed Care (VDC) program from 71 to all 171 Veterans Affairs Medical Centers (VAMCs) by the end of fiscal year 2024. VDC enables nursing-home eligible Veterans to remain at home and work with Aging and Disability Network Agencies (ADNAs) to coordinate personalized care services and supports. Enrolled Veterans receive an annual budget to hire caregiver employees, often family and friends, and acquire other needed services. Between October 2024 to June 2025, we conducted 55 phone interviews with VA and ADNA program coordinators and staff, Veterans, and caregivers across 10 VDC programs nationwide, including six expansion sites and four established programs. Interviews were recorded, transcribed, and analyzed to identify barriers and facilitators to VDC implementation. Staff data were analyzed using the Consolidated Framework for Implementation Research (CFIR) 2.0, and thematic analysis was applied to Veteran and caregiver data. We found that VDC is highly recipient-centered, focused on Veterans’ needs and promotes self-management and aging in place. Facilitators included making VDC a VA performance metric, staff motivation, clear communication and shared missions between VAMCs and ADNAs, and flexible tailoring to each site’s context. Program complexity emerged as a barrier due to varying VDC employer models, multi-state and regional coverage requiring different processes, inadequate staffing, and limited rural coverage. Challenges for Veterans included administrative management for employing caregivers. The evaluation identified factors impacting VDC implementation that can inform VA-community partnerships to improve Veteran-centered care delivery.

